# Cichoric Acid Ameliorates Monosodium Urate-Induced Inflammatory Response by Reducing NLRP3 Inflammasome Activation via Inhibition of NF-*k*B Signaling Pathway

**DOI:** 10.1155/2021/8868527

**Published:** 2021-01-06

**Authors:** Qi Wang, Bingfeng Lin, Zhifeng Li, Jie Su, Yulin Feng

**Affiliations:** ^1^Jiangxi University of Traditional Chinese Medicine, Nanchang 330002, China; ^2^Nanchang Key Laboratory of Active Ingredients of Traditional Chinese Medicine and Natural Medicine, Nanchang 330006, China; ^3^State Key Laboratory of Innovative Drug and Efficient Energy-Saving Pharmaceutical Equipment, Nanchang 330006, China

## Abstract

Gouty arthritis is characterized by the deposition of monosodium urate (MSU) within synovial joints and tissues due to increased urate concentrations. Here, we elucidated the role of the natural compound cichoric acid (CA) on the MSU crystal-stimulated inflammatory response. The THP-1-derived macrophages (THP-Ms) were pretreated with CA and then stimulated with MSU suspensions. The protein levels of p65 and I*κ*B*α*, the activation of the NF-*κ*B signaling pathway by measuring the expression of its downstream inflammatory cytokines, and the activity of NLRP3 inflammasome were measured by western blotting and ELISA. CA treatment markedly inhibited the degradation of I*κ*B*α* and the activation of NF-*κ*B signaling pathway and reduced the levels of its downstream inflammatory genes such as IL-1*β*, TNF-*α*, COX-2, and PGE2 in the MSU-stimulated THP-M cells. Therefore, we infer that CA effectively alleviated MSU-induced inflammation by suppressing the degradation of I*κ*B*α*, thereby reducing the activation of the NF-*κ*B signaling pathway and the NLRP3 inflammasome. These results suggest that CA could be a novel therapeutic strategy in averting acute episodes of gout.

## 1. Introduction

Gout, one of the most severe and common forms of inflammatory arthritis, results from hyperuricemia and deposition of monosodium urate (MSU) crystals in articular joints and periarticular tissues [1]. In China, epidemiological studies found that the corresponding rates of hyperuricemia and gout are 13.3% and 1.1%, accompanied by increased dietary consumption of purine-rich food and alcohol [2]. Currently, nonsteroidal anti-inflammatory drugs (NSAIDs) and colchicine are the most frequently used treatments, but their usage is restricted due to the inevitable gastrointestinal or cardiovascular side effects. In the circumstances of NSAID contraindications, interleukin (IL)-1 inhibitors and steroids, such as glucocorticoids, can be used but their usage is also limited by high cost and hematopoietic disorders [3]. Therefore, it is crucial to develop novel agents for the treatment of gouty arthritis.

Gout is triggered by the deposition of monosodium urate (MSU) crystals in and around joints where macrophages play a critical role in its occurrence, growth, and regression [4, 5]. Based on distinct stimuli, macrophages can polarize into two extreme phenotypes: the classically activated M1-like phenotype and the alternatively activated M2-like phenotype [6]. Polarization into the M1 phenotype promotes the production of proinflammatory cytokines IL-1*β* and TNF-*α* that further aggravate gout [7]. Also, prostaglandin E2 (PGE2), converted from arachidonic acid (AA) by cyclooxygenase (COX), contributes to the production of IL-1*β*, an important mediator in gout [8–10]. Therefore, several cyclooxygenase-2 (COX-2) inhibitors have been clinically used to treat gouty arthritis [11]. Previous studies showed that NF-*κ*B (nuclear factor kappa B) and NLRP3 (NLR family pyrin domain containing 3) signaling pathways are associated with MSU crystal-induced inflammatory cytokine release in the monocytes/macrophages. Particularly, priming of the NLRP3 inflammasome is dependent on NF-*κ*B [12]. Activation of NLRP3 inflammasome promotes MSU crystal-induced inflammation in macrophages, while its inactivation averts gout by reducing the production of IL-1*β* [13]. Thus, diminishing the production of IL-1*β* or TNF-*α* in macrophages by inhibiting the M1 polarization and/or NLRP3 inflammasome activation via the NF-*κ*B signal pathway could be an effective strategy to treat gouty arthritis.

Cichoric acid (CA), isolated from edible plant species of the Asteraceae family *Cichorium intybus*, is a derivative of caffeic and tartaric acid and is also found in *Bidens tripartita* L., *Echinacea purpurea*, and *Cichorium endivia* L. [14]. Several recent studies reported that cichoric acid has significant pharmacological properties such as antitumor, free radical scavenging, and anti-inflammatory [15–18]. *Cichorium intybus* significantly reduces the uric acid, and the effect has been related to CA, the key content of this Chinese herb [19]. Furthermore, in HepG2 cells, CA was shown to promote glucosamine-mediated glucose uptake and inhibit inflammation through NF-*κ*B signaling pathways [20]. In synovial tissues of the ankle joint, CA could significantly decrease the levels of nuclear factor-*κ*B (NF-*κ*B), TNF-*α*, and cyclooxygenase-2 (COX-2) [21]. Therefore, we hypothesized that CA could be explored for the treatment of acute gout, induced by MSU through the NF-*κ*B signaling pathway.

In this study, we show that CA can inhibit MSU crystal-stimulated inflammation in THP-M cells by diminishing the levels of IL-1*β*, TNF-*α*, COX-2, PGE2, cAMP, and PKA by inhibiting the I*κ*B*α* degradation and reducing the activation of the NF-*κ*B signaling pathway and NLRP3 inflammasome. Overall, these findings set the basis for novel gout arthritis therapy.

## 2. Materials and Methods

### 2.1. Materials

Highly pure (>98%) CA was obtained from the National Institutes for Food and Drug Control (Beijing, China). THP-1 cell line was purchased from American Tissue Culture Collection (ATCC, Rockville, MD, USA). The specifics of the other materials used in the study are as follows: COX-1 Inhibitor Screening Kit (Fluorometric) (BioVision, Inc., Mountain View, CA, USA); COX-2 Inhibitor Screening Kit (Beyotime Biotech Co., Ltd., China); PVDF (0.45 *μ*m) (Millipore, Schwalbach, Germany); precolor protein marker (Green BioResearch, LA, USA); fuchsia, Tween 20, acrylamide, and sodium dodecyl sulfate (Solon, OH, USA); protein lysate (RIPA) (Beyotime Biotechnology, Shanghai, China); western blotting membrane regeneration solution (C500031, Sangon Biotech, Shanghai, China); fetal bovine serum (FBS) (GIBCO, NY, USA); RPM1-1640 culture medium (GIBCO, NY, USA); trypsin (Gibco, Grand Island, NY, USA); phorbol myristate acetate (PMA) and penicillin (Sigma-Aldrich, St. Louis, MO); TNF-*α* ELISA kit (DTA00D) and IL-1*β* ELISA kit (DLB50) (R&D Systems, Minnesota, USA); anti-CD86 antibody (ab77276), anti-TNF-*α* antibody (ab269282), anti-NLRP3 antibody (ab263899), PKA kinase activity assay kit (ab139435), Prostaglandin E2 ELISA Kit (ab133021), and cAMP assay kit (Competitive ELISA) (ab234585) (Abcam, Cambridge, UK); anti-IL-1*β* antibody (12703), anti-phos-p65 (Ser536) antibody (3033), anti-p65 antibody (8242), anti-phos-I*κ*B*α* (Ser32) antibody (2859), anti-I*κ*B*α* antibody (9242), anti-COX-2 antibody (12282) (CST, Boston, USA), and TRIzol (Invitrogen); reverse transcription kit (1708843) and SsoFast EvaGreen Supermix (1725200) (Bio-Rad Laboratories, California, USA); primer (Sangon Biotech Co., Ltd. Shanghai, China); BD Cytofix/Cytoperm™ Fixation/Permeabilization Kit (554714) (BD, New Jersey, USA); ionomycin (SQ23377) (MCE, Shanghai, China); MSU (Invivogen, France); Berthold LB941 microporous plate-type multifunctional enzyme labeling instrument; flow cytometry (Beckman DxFLEX, USA); real-time fluorescence quantitative PCR instrument (ABI 7500, ABI, USA).

### 2.2. Cell Culture and MSU Treatment

THP-1 cells were cultured in RPMI 1640 containing 10% fetal bovine serum, 0.05 mM 2-mercaptoethanol, 100 U/ml penicillin, and 100 *μ*g/ml streptomycin. These were then seeded (1 × 10 ^5^ cells/ml/well) into 6-well culture plates. As described previously, THP-1-derived macrophages (THP-Ms) were obtained by treating the cells with 150 ng/ml PMA for 24 h [22]. Then, after pretreatment with different concentrations of cichoric acid (30 *μ*g/ml, 100 *μ*g/ml, and 300 *μ*g/ml), THP-Ms were subjected to MSU suspension (1.0 mg/ml) for 24 h. The supernatants were used to detect the cytokine levels. ELISA, as per the manufacturer's guidelines, was used to determine the levels of IL-1*β*, TNF-*α*, PGE2, and cAMP. The activity of PKA (protein kinase A) was determined using a multifunctional enzyme marker.

### 2.3. Western Blotting

Total protein extracts were prepared from the THP-M cells using the RIPA buffer. Cytoplasmic Protein Extraction Kit (Beyotime, China) was used to extract the cytosolic proteins. The protein concentrations were determined using the BCA protein assay kit (Thermo Scientific, MA, USA). The protein samples were separated by SDS-PAGE (dodecyl sulfate-polyacrylamide gel electrophoresis) and transferred onto PVDF (polyvinylidene difluoride) membranes. After blocking, the membranes were incubated with the corresponding primary antibodies (1 : 1000) at 4°C overnight. Then, the incubation with secondary antibodies (1 : 5000) was carried out for 1 h at room temperature (RT). Finally, using a chemiluminescence kit, the intensity of protein bands was detected by the gel imaging system, and the ImageJ software was used for the quantitative measurements.

### 2.4. RNA Extraction and Real-Time Quantitative PCR (qPCR) Analysis

Using the TRIzol method, total RNA extracted from the THP-1 cells was reverse-transcribed into cDNA [23]. The expression levels of CD86 and iNOS were analyzed using the SYBR green gene expression assay. The corresponding primer sequences are presented in Table 1.

### 2.5. Flow Cytometry

The cell suspension concentration was adjusted to 1 × 10^6^ cells/ml. Like previously, cells were then pretreated with CA (30 *μ*g/ml, 100 *μ*g/ml, and 300 *μ*g/ml) [22]. CD86 and TNF-*α* antibodies were added and incubated in dark for 30 min at RT. Finally, the cells were washed with PBS and BD Perm/Wash buffer and resuspended into 500 *μ*l ice-cold PBS for the flow cytometry experiments.

### 2.6. Statistical Analysis

All values are expressed as mean ± SEM. Statistical analyses were performed using the one-way ANOVA, and the multiple group comparisons were carried out using the Student–Newman–Keul test. Statistical significance of changes over time was evaluated by one-way repeated measures ANOVA, followed by Bonferroni's post hoc test. *p* values ≤ 0.05 were considered statistically significant.

## 3. Results

### 3.1. CA Inhibits the Polarization of THP-M Cells

**Figure 1:**
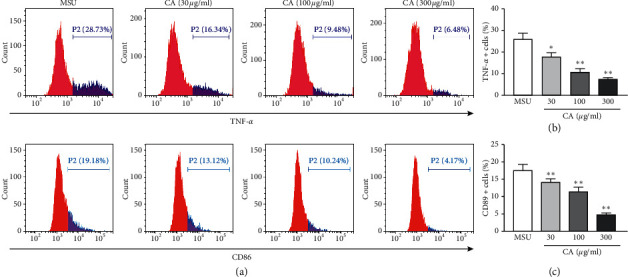
The polarization of the distinctly treated THP-M cells was evaluated by flow cytometry. Flow cytometric analysis for cluster of differentiation in the corresponding THP-M cells expressing TNF-*α* and CD86 (a), statistical analysis (b, c). The results are presented as mean ± SD (*n* = 3). ^*∗∗*^*p* < 0.01 vs. the MUS group.

**Figure 2:**
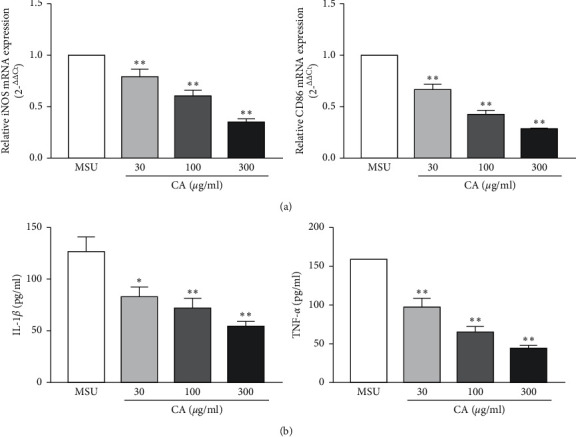
Effect of CA on the mRNA levels of iNOS and CD86 in the THP-M cells. The expression levels of iNOS and CD86 were detected by RT-PCR. The mRNA levels of iNOS and CD86 were normalized to MUS (a). Inhibitory effects of CA on IL-1*β* and TNF-*α* secretion in the THP-M cells. The culture medium was collected, and the levels of IL-1*β* and TNF-*α* were determined by ELISA (b). The results are presented as mean ± SD (*n* = 3). ^*∗*^*p* < 0.05, ^*∗∗*^*p* < 0.01 vs. the MUS group.

Firstly, to examine if CA would modulate macrophage phenotype switch, we assessed the macrophage polarization status in THP-M cells. The high-level expression of iNOS, CD86, and TNF-*α* is an important attribute of the M1-like macrophages. Therefore, we carried out the flow cytometry analysis for cluster of differentiation for TNF-*α* and CD86 in THP-M cells. In our preliminary experiment (sFigure 1), we have evaluated the polarization of different concentrations treated THP-M cells and the result showed that the dose of CA was 1–10 *μ*g/ml; there was no significant effect on the polarization of macrophages, and when the dose of CA was more than or equal to 30 *μ*g/ml, the polarization of macrophages was significantly reduced. Compared with 300 *μ*g/ml, 600 *μ*g/ml had no significant effect on the polarization of macrophages. Therefore, the concentration of CA was 30 *μ*g/ml, 100 *μ*g/ml, and 300 *μ*g/ml, respectively. As shown in Figure 1, compared with the MSU group, the CA (30 *μ*g/ml, 100 *μ*g/ml, and 300 *μ*g/ml) group showed a significantly decreased level of M1 polarization. Furthermore, upon qPCR analysis of mRNA expression levels of iNOS and CD86, as expected, we found that compared to the MSU group, the expression of iNOS and CD86 was lowered in the CA group of THP-M cells (Figure 2(a)).

### 3.2. Effects of CA on NLRP3 Inflammasome in the THP-M Cells

**Figure 3:**
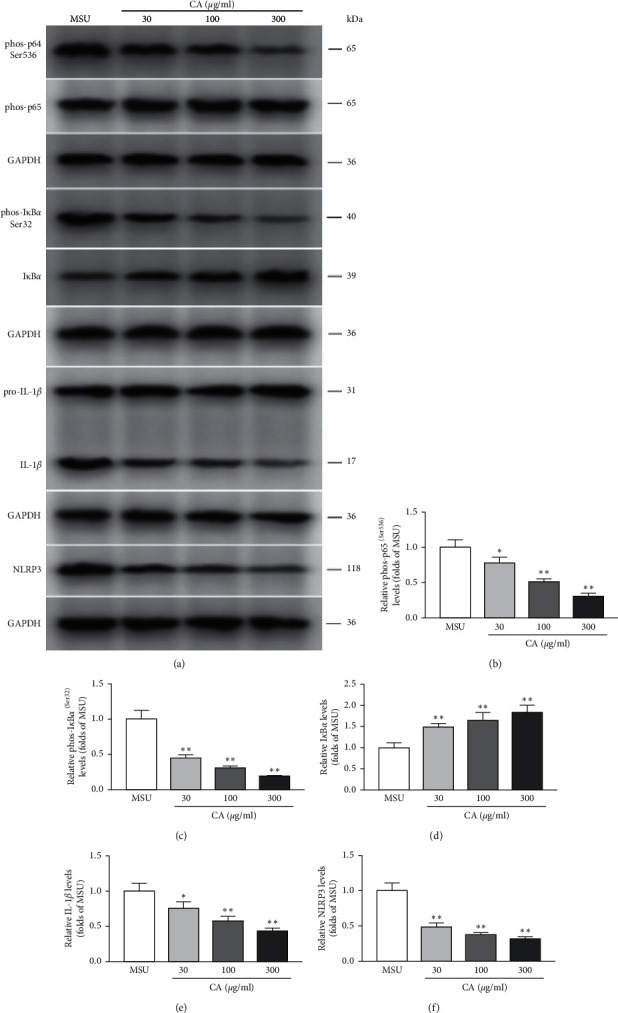
Relative levels of phosphorylation of multiple proteins in the THP-M cells. The protein levels of p65, IL-1*β*, pro-IL-1*β*, I*κ*B*α*, and NLRP3 and the levels of phosphorylated p65 (Ser536) and p-I*κ*B*α* (Ser32) from different groups were analyzed by western blotting (a). The protein levels of phos-p65 (Ser536) (b), phos-I*κ*B*α* (Ser32) (c), I*κ*B*α* (d), IL-1*β* (e), and NLRP3 (f) were normalized to MUS. The results are presented as mean ± SD (*n* = 3). ^*∗∗*^*p* < 0.01 vs. the MSU group.

**Figure 4:**
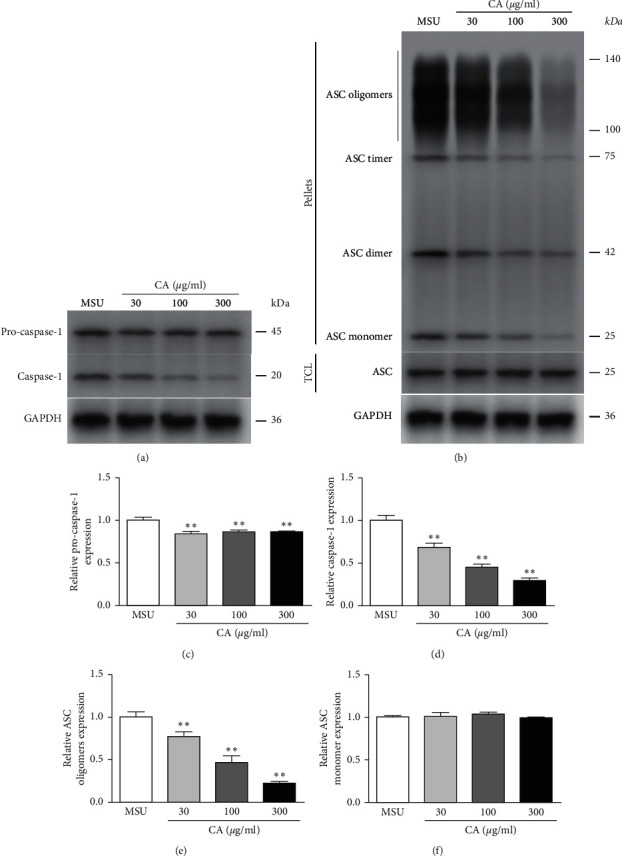
Effect of CA on ASC and caspase-1 in the THP-M cells. The protein levels of pro-caspase-1, caspase-1, ASC oligomers, and ASC monomer from different groups were analyzed by western blotting (a, b). The protein levels of pro-caspase-1 (c), caspase-1 (d), ASC oligomers (e), and ASC monomer (f) were normalized to MUS. The results are presented as mean ± SD (*n* = 3). ^*∗∗*^*p* < 0.01 vs. the MSU group.

**Figure 5:**
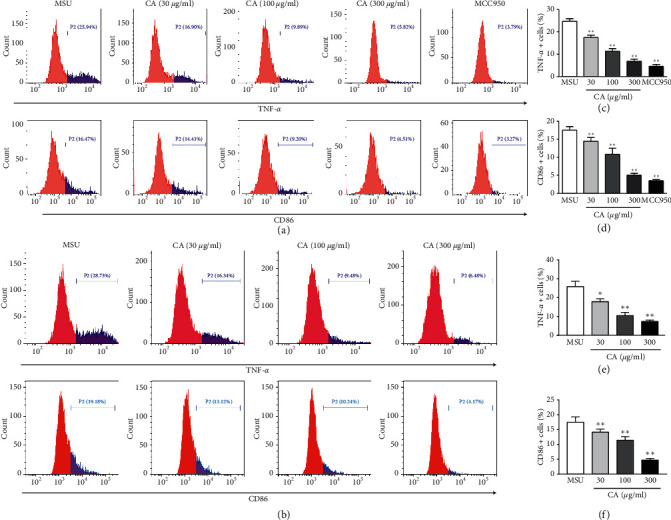
The polarization of the distinctly treated THP-M cells was evaluated by flow cytometry. Flow cytometric analysis for cluster of differentiation in the corresponding THP-M cells expressing TNF-*α* and CD86 (a, d). Statistical analysis (b) (c), (e), and (f). The results are presented as mean ± SD (*n* = 3). ^*∗∗*^*p* < 0.01 vs. the MUS group.

**Figure 6:**
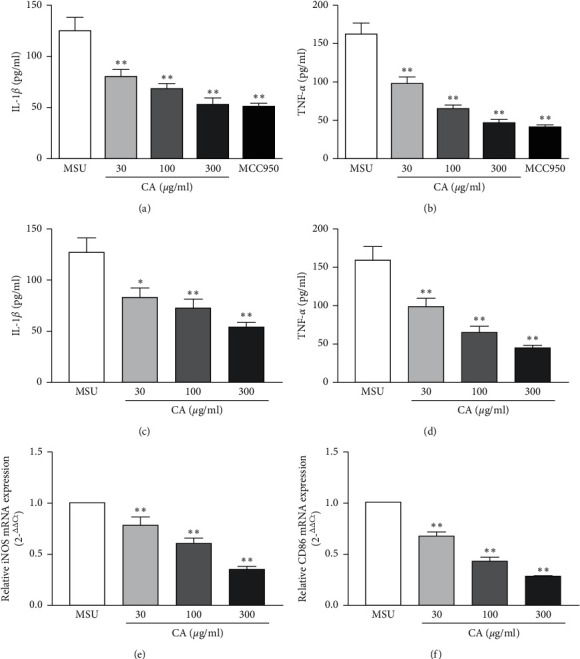
Inhibitory effects of CA on IL-1*β* and TNF-*α* secretion in the THP-M cells. The culture medium was collected, and the levels of IL-1*β* (a, c) and TNF-*α* (b, d) were determined by ELISA. Effect of CA on the mRNA expressions of iNOS and CD86 in THP-M cells. The mRNA levels of iNOS (e) and CD86 (f) were detected by RT-PCR. The results are presented as mean ± SD (*n* = 3). ^*∗*^*p* < 0.05, ^*∗∗*^*p* < 0.01 vs. the MUS group.

Next, we investigated the effect of CA on NLRP3 inflammasome in the THP-M cells. For this, enzyme-linked immunosorbent assay (ELISA) was used to measure the levels of proinflammatory cytokines, IL-1*β*, and TNF-*α*. We found that CA could suppress the MSU-induced levels of IL-1*β* and TNF-*α*. Moreover, the effects were also consistent in a dose-dependent manner (Figure 2(b)). Since functional and mature IL-1*β* is cleaved by cytosolic inflammasome from its precursor, the total protein samples were subjected to western blotting to analyze the levels of key components of the NLRP3 inflammasome. We found that MSU dramatically increased the levels of NLRP3, IL-1*β*, caspase-1, ASC oligomers, and ASC monomer; however, CA could reduce them in a dose-dependent manner (Figures 3(e), 3(f), and 4). Furthermore, we investigated the effect of CA treatment comparing in parallel with NLRP3 inflammasome inhibitors, MCC950, and the result indicated that compared with MSU group, the levels of IL-1*β*, TNF-*α*, and CD86 on macrophage surface in the CA group and positive drug MCC950 group were significantly decreased; that is, macrophage polarization level was significantly reduced in a dose-dependent manner. The best effect was obtained at 300 *μ*g/ml, which was close to the efficacy of positive drug (Figures 5 and 6). Overall, these results indicate that CA is capable of suppressing the production of MSU-induced proinflammatory cytokines by reducing the activation of the NLRP3 inflammasome.

### 3.3. Effects of CA on NF-*κ*B Signaling

A previous study, in HepG2 cells, showed that CA promotes glucosamine-mediated glucose uptake and inhibits inflammation through NF-*κ*B signaling pathways. Here, also we observed the CA effects on the MSU-stimulated upregulation of the key components of the inflammasome in macrophages; therefore, we speculated that CA-mediated effects could be via regulation of the NF-*κ*B signaling pathway. To verify this, we examined the protein levels of p65, p-p65, I*κ*B-*α*, and p-I*κ*B-*α* by western blotting. We found that CA, dose-dependently, suppressed the MSU-induced upregulation of p-p65 and p-I*κ*B-*α* and therefore increased the levels of p65 and I*κ*B-*α*, correspondingly (Figure 3). These results indicate that CA reduced MSU crystal-induced inflammation certainly via suppressing NF-*κ*B signaling. In these experiments, GADPH was used as the internal control.

### 3.4. Effects of CA on the Levels of cAMP, PKA, PGE2, and COX-2

**Figure 7:**
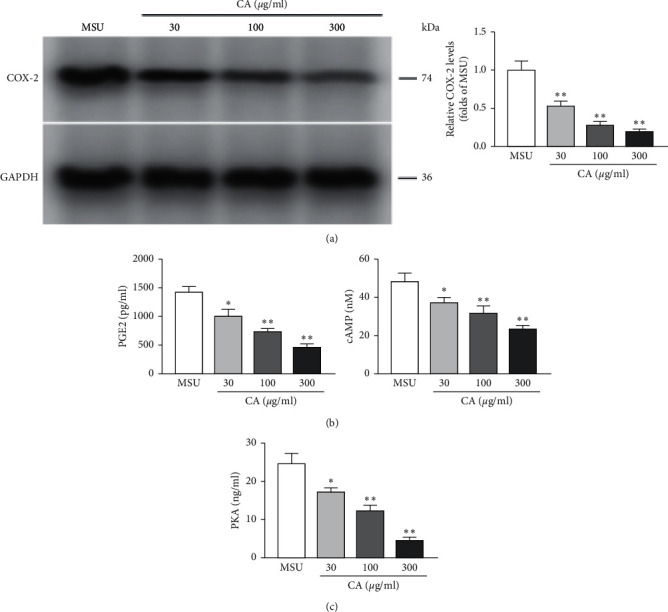
Effect of CA on COX-2 levels in the THP-M cells. After the indicated treatment, the levels of COX-2 among distinct groups were detected by western blotting. The level of COX-2 was normalized to MUS (a). Inhibitory effects of CA on PGE2 and cAMP in the THP-M cells. The levels of PGE2 and cAMP were determined by ELISA (b). Inhibitory effects of CA on PKA in the THP-M cells (c). The results are presented as mean ± SD (*n* = 3). ^*∗*^*p* < 0.05, ^*∗∗*^*p* < 0.01 vs. the MUS group.

We further investigated the effect of CA on COX-2, PGE2, cAMP, and PKA in the THP-M cells. The levels of COX-2 were determined by western blotting (Figure 7(a)), PGE2 and cAMP were determined by ELISA (Figure 7(b)), and PKA activity was determined by the BCA kit (Figure 7(c)), respectively. We found that compared with the MSU group, the CA group (30 *μ*g/ml, 100 *μ*g/ml, and 300 *μ*g/ml) of cells showed significantly decreased levels of COX-2, PGE2, cAMP, and PKA. This indicates that CA reduced MSU crystal-induced inflammation by suppressing MSU-induced increase of COX-2, PGE2, cAMP, and PKA.

## 4. Discussion

In this study, for the first time, we demonstrated that CA alleviates the MSU crystal-induced inflammation by altering the macrophage polarization away from the M1-like phenotype. Moreover, CA suppresses the levels of p-p65 and p-I*κ*B*α*, mediating the activation of the NLRP3 inflammasome, reducing the levels of IL-1*β*, TNF-*α*, COX-2, cAMP, PKA, and PGE2 via the NF-*κ*B signaling pathway.

THP-M cells were obtained by PMA treatment from the THP-1 cells. Under normal circumstances, both M1 and M2 phenotypes are observed in the THP-Ms. However, the polarization of macrophages toward the proinflammatory M1 phenotype exacerbates gout attacks [7]. M1-like macrophages are characterized by high expression of iNOS, CD86, and TNF-*α*. Here, using flow cytometry, we observed that CA treatment skewed macrophage polarization away from the M1-like phenotype by reducing the levels of the aforementioned cytokines.

The NLRP3 inflammasome, an important member of the innate immune system, regulates the release of inflammatory cytokines, which are activated by various stimuli such as bacterial toxins and crystals [24, 25]. An important feature of gouty arthritis is the activation of NLRP3 inflammasome and the release of IL-1*β* [26]. Especially, in M1-polarized macrophages, the NLRP3 inflammasome is highly activated [27] and critically plays an important role in the MSU crystal-induced inflammatory response. Notably, the strategies that could either block or reduce the activation of NLRP3 inflammasome could reduce gout inflammation [28]. Han et al. reported that dioscin inhibited the activation of NLRP3 through downregulating the protein expressions of NLRP3, apoptosis-associated speck-like protein containing a caspase recruitment domain (ASC), cleaved-caspase-1, and IL-1*β* [29]. Yin et al. also found that eucalyptol acts against monosodium urate-mediated gouty arthritis through inhibiting inflammasome NLRP3 [30]. In addition, Wang et al. discovered that chicory extract had antigout inflammatory effect by suppressing the NF-*κ*B and NLRP3 signaling pathways in gout rats and verified the main component cichoric acid may be the main active component [31]. However, he did not conduct in-depth research on the effect of cichoric acid. So, in this study, we mainly focused on the role and mechanism of cichoric acid and found that CA also functions by suppressing the activation of MSU crystal-induced NLRP3 inflammasome. These results suggest that NLRP3 inflammasome may be an important therapeutic target for gout arthritis.

The activation of the NF-*κ*B signaling pathway is central in several inflammation-related diseases, including gouty arthritis [32]. In the proteasome, the degradation of inhibitory protein I*κ*B*α* leads to the activation of the NF-*κ*B signaling pathway which then regulates downstream genes, such as proinflammatory cytokines IL-1*β*, IL-6, and TNF-*α* [33, 34]. Disorderly NF-*κ*B activation is also closely associated with MSU crystal-induced inflammation [14]. Measuring the expression of NF-*κ*B signaling pathway-related genes could be an important tool for studying CA therapeutic effects in gout arthritis. Here, we first evaluated the anti-inflammatory effects of CA in MSU crystal-induced inflammation in the THP-M cells. Two critical proteins of the NF-*κ*B signaling pathway were analyzed using western blotting. Notably, after CA treatment, the level of I*κ*B*α* was increased; however, phos-I*κ*B*α* levels decreased correspondingly, in a dose-dependent manner. Also, the protein levels of p-p65 were significantly reduced, indicating that CA attenuated NF-*κ*B activation. Since expression levels of IL-1*β*, TNF-*α*, COX-2, and PGE2 are regulated by NF-*κ*B signaling [35–37], we also observed that CA treatment reduced the levels of IL-1*β*, TNF-*α*, COX-2, and PGE2 in a dose-dependent manner. It is likely that CA alleviated the MSU-induced inflammation via NF-*κ*B signaling.

During an inflammatory response, COX-2 is activated by external stimuli, which augments the synthesis of PGE2 from arachidonic acid. Then, PGE2 activates PKA by upregulating the level of cAMP through the EP receptor, triggering the downstream NF-*κ*B pathway. Furthermore, the COX-2 gene is also one of the target genes of the NF-*κ*B signaling pathway; this forms a positive feedback mechanism that magnifies the inflammatory response. Apart from inhibiting the level of COX-2, PGE2, cAMP, and PKA, we envisage that CA can also impede the aforesaid inflammatory positive feedback, increasing its effect in gout treatment.

## 5. Conclusion

In conclusion, we show that CA, a widely used natural compound in *Cichorium intybus* with various biological properties, effectively prevented the MSU crystal-induced inflammatory response in macrophages. The underlying mechanisms of CA effects are associated with the modulation of NLRP3 inflammasome via the NF-*κ*B signaling pathway. Overall, these results suggest that CA could be a promising therapeutic agent for the prevention and treatment of gouty arthritis in the clinic.

## Figures and Tables

**Table 1:** Primers used in real-time quantitative PCR.

Primers		Sequence (5′⟶3′)
iNOS	Forward	5-CGCATGACCTTGGTGTTTGG-3
	Reverse	5-CATAGACCTTGGGCTTGCCA-3

CD86	Forward	5- CCAAAATGGATCCCCAGTGC-3
	Reverse	5- AAGTTAGCAGAGAGCAGGAAGG-3

GAPDH	Forward	5- GAAGGTGAAGGTCGGAGTC-3
	Reverse	5- GAAGATGGTGATGGGATTTC-3

## Data Availability

The data used to support the findings of this study are available from the corresponding author upon reasonable request.

## Supplementary Materials

Supplementary Materials The polarization of the distinctly treated THP-M cells was evaluated by flow cytometry.
