# Supplementary material for: Cichoric Acid Ameliorates Monosodium Urate-Induced Inflammatory Response by Reducing NLRP3 Inflammasome Activation via Inhibition of NF-kB Signaling Pathway
Source: Evid Based Complement Alternat Med. 2021 Jan 6;2021:8868527. doi: 10.1155/2021/8868527 (PMC7808822; doi:10.1155/2021/8868527)
Supplement: Supplementary Materials — The polarization of the distinctly treated THP-M cells was evaluated by flow cytometry. [file 8868527.f1.docx]

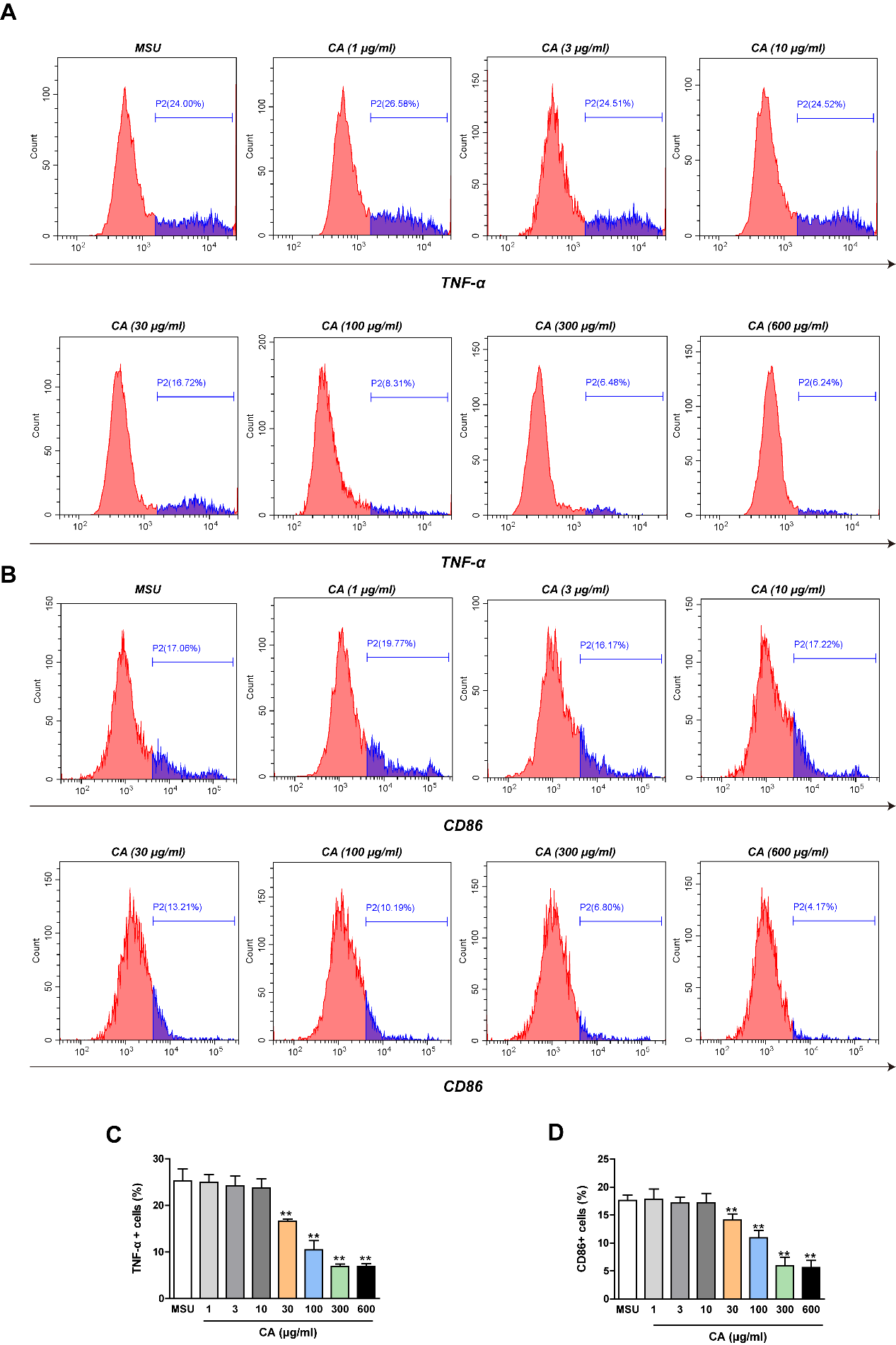


sFig. 1. The polarization of the distinctly treated THP-Ms cells was evaluated by flow cytometry. Flow Cytometric Analysis for Cluster of Differentiation in the corresponding THP-Ms cells expressing TNF-α and CD86 (A and B). Statistical analysis (C and D). The results are presented as mean ± SD (n = 3). **p <0.01 vs. MUS group.
